# Cytoplasmic incompatibility factor proteins from *Wolbachia* prophage are costly to sperm development in *Drosophila melanogaster*

**DOI:** 10.1098/rspb.2024.3016

**Published:** 2025-02-12

**Authors:** Rupinder Kaur, Seth R. Bordenstein

**Affiliations:** ^1^ Departments of Biology and Entomology, Pennsylvania State University, University Park, PA, USA; ^2^ One Health Microbiome Center, Pennsylvania State University, Huck Institutes of the Life Sciences, University Park, PA, USA

**Keywords:** *drosophila *spermatogenesis, spermiogenesis, sperm shape defects, *wolbachia *symbiosis, cytoplasmic incompatibility, prophage cif proteins

## Abstract

The symbiosis between arthropods and *Wolbachia* bacteria is globally widespread, largely due to selfish-drive systems that favour the fitness of symbiont-transmitting females. The most common drive, cytoplasmic incompatibility (CI), is central to arboviral control efforts. In *Drosophila melanogaster* carrying *w*Mel *Wolbachia* deployed in mosquito control, two prophage genes in *Wolbachia, cifA* and *cifB*, cause CI that results in a paternal-effect lethality of embryos in crosses between *Wolbachia*-bearing males and aposymbiotic females. While the CI mechanism by which Cif proteins alter sperm development has recently been elucidated in *D. melanogaster* and *Aedes aegypti* mosquitoes, the Cifs’ extended impact on male reproductive fitness such as sperm morphology and quantity remains unclear. Here, using cytochemical, microscopic and transgenic assays in *D. melanogaster,* we demonstrate that both CifA and CifB cause a significant portion of defects in elongating spermatids, culminating in malformed mature sperm nuclei. Males expressing Cifs have reduced spermatid bundles and sperm counts, and transgenic expression of Cifs can occasionally result in no mature sperm formation. We reflect on Cifs’ varied functional impacts on the Host Modification model of CI as well as host evolution, behaviour and vector control strategies.

## 1. Background

*Wolbachia* are maternally inherited, obligate intracellular bacteria that occur worldwide in 40–65% of arthropod species [1]. Many *Wolbachia* strains selfishly alter host reproductive biology to increase the relative number of symbiotic females that vertically transmit the bacteria to the next generation [2]. Among these reproductive alterations, the most common and relevant to vector control is cytoplasmic incompatibility (CI). CI is a sperm–egg incompatibility that results in embryonic mortality when modified sperm from *Wolbachia* symbiotic males fertilize eggs from incompatible, aposymbiotic females. The reciprocal crosses with symbiotic females are compatible [3]. *Wolbachia* thus selfishly increase the fitness of symbiotic females and their offspring, thereby promoting their own maternal spread.

At the genetic level, CI in *Drosophila melanogaster* is caused by testes expression of two genes*—cifA* and *cifB*—encoded in the prophage of *w*Mel *Wolbachia* [4,5]. We recently reported in *D. melanogaster* and *Aedes aegypti* mosquitoes that *w*Mel and transgenically-expressed Cifs establish CI via the Host Modification model by altering sperm chromatin integrity. Specifically, Cifs deplete long non-coding RNA (lncRNA) in spermatocytes, nick DNA in elongating spermatids and disrupt the histone-to-protamine (H–P) transition essential for determining sperm fertility [6–8]. It remained unclear whether, apart from inducing CI, Cifs impose a fitness cost on host sperm biology. Here we show that in addition to CI, the *w*Mel and Cif proteins cause shape defects in elongating and mature sperm nuclei in *D. melanogaster*. Moreover, flies suffer from reduced and, in some cases, null sperm count, suggesting that Cifs are costly to sperm development. We reflect on the impact of these findings on host evolution, behaviour and ongoing vector control strategies.

## 2. Results

### (a) *Wolbachia* and Cifs cause shape defects during the histone-to-protamine transition stage of spermiogenesis

**Figure 1.**
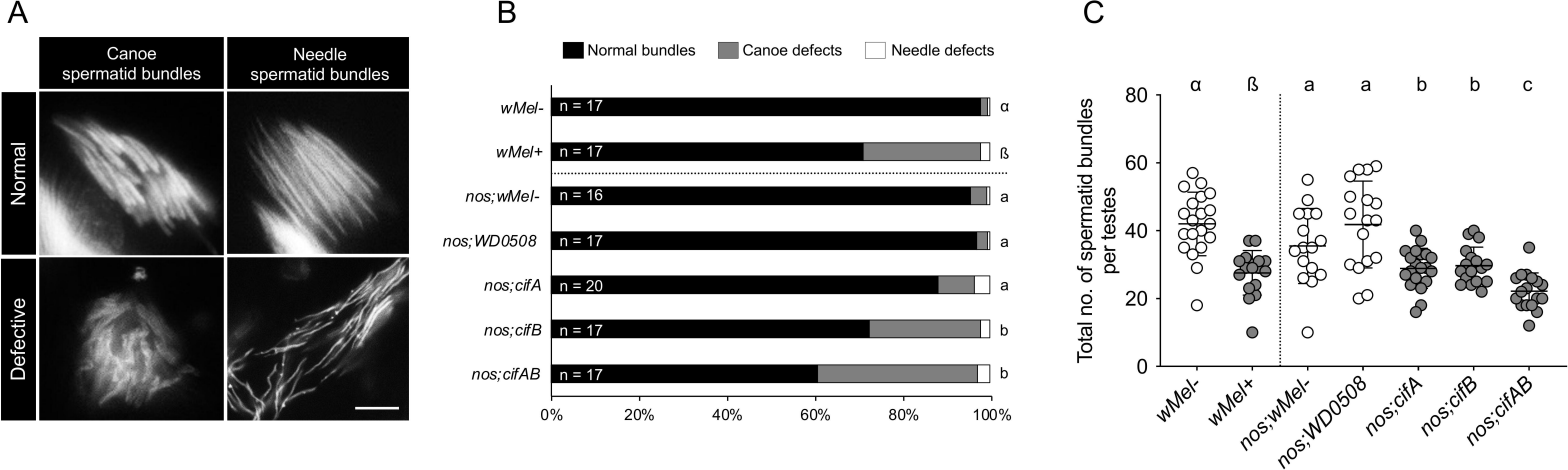
*Wolbachia* and Cifs cause shape defects during the histone-to-protamine transition stage of spermiogenesis. Testes (*n* = 15–20) from <8 h old males of CI-causing, wild type *w*Mel+ and transgenic *cifAB* lines were dissected and stained with DAPI stain to label spermatid DNA. Single transgene-expressing lines *cifA*, *cifB*, and *WD0508* were used as non-CI controls. To control for any background confounding effects of the *nos-Gal4:VP16* driver line, *w*Mel− males were crossed to *nos* females to generate males with a *nos;wMel−* genotype. (A) Representative images of defective versus normal spermatid head morphology are shown at the canoe and needle stages of spermiogenesis. Scale bar: 10 µm. (B) Both *w*Mel+ and *cifAB* males showed higher percentage of defective canoe-spermatid bundles compared with negative controls. *cifB* induced higher damage than *cifA*. (C) Total spermatid bundles including both canoe and needle stages were manually counted and graphed. Both *w*Mel+ and transgenic *cif*-expressing males showed significantly less bundles compared with negative controls. Vertical bars represent mean, and error bars represent standard deviation. Letters indicate statistically significant (*p* < 0.05) differences as determined by a pairwise comparison using Mann–Whitney *U*-test between *w*Mel+ and *w*Mel-, and multiple comparisons based on a Kruskal–Wallis test and Dunn multiple test correction among transgenic groups. All *p*-values are reported in electronic supplementary material, table S1.

In *D. melanogaster* flies and *Ae. aegypti* mosquitoes, CifA and CifB proteins from *w*Mel initially localize to developing sperm DNA from the pre-meiotic spermatocytes onwards to elongated spermatids [6,8]. Here, using the 4',6-diamidino-2-phenylindole (DAPI) DNA stain in *D. melanogaster* wild type and dual *cifAB*-expressing testes, we report that the first visible sperm head shape defects arise during the canoe and needle elongating stages (figure 1A). Notably, the canoe stage is when the Cifs elevate DNA nicking and alter the H–P transition. Specifically, portions of heads in canoe spermatid bundles were distorted in shape, and some needle spermatid bundles were found loosely packed. When sperm bundles were aberrant, all visible spermatids present in those cyst bundles were found defective compared with unaltered spermatids in other bundles and the normal wild-type bundles that develop with regular shape. CI-causing *w*Mel+ males (*Wolbachia* present) exhibited defects in 28.8% of total bundles spanning canoe stage (26.7% of total bundles with misshaped heads) and needle stage (2.1% of loosely packed bundles). In contrast, *w*Mel− bundles (*Wolbachia* absent) were normal in shape (97.9%) with only 1.7% defective bundles at canoe stage and 0.5% at needle stage (figure 1B; electronic supplementary material, figures S1, table S1). Moreover, *w*Mel+ males made 32.1% fewer spermatid bundles per testes, in total (figure 1C).

Transgenic CI flies dually expressing *cifA* and *cifB* similarly harboured 60.8% normal sperm, 36.4% shaped defects at the canoe stage and 2.8% packaging defects at the needle stage relative to the negative control counterparts of (i) nos; *w*Mel− males that had 95.7% normal spermatids with only 3.7% canoe and 0.7% needle defects and (ii) nos; *WD0508* [4] males that had 97% normal sperm with 2.5% canoe and 0.5% needle defects. Upon single transgenic expression, both *cifA-* and *cifB-*expressing males had a similar number of total bundles per testes; however, *cifB* induced stronger defects at the canoe stage (25.4%) than *cifA* (8.3%; figure 1B; electronic supplementary material, figures S1, table S1), possibly due to *cifB*’s enhanced nuclease activity at the spermiogenesis stage [7]. Once again, single *cifA* or *cifB* and dual *cifAB* males made approximately 30–45% fewer sperm bundles than the control (figure 1C), indicating that the Cifs alone or together can negatively impact sperm development, causing phenotypes ranging from CI to these shape defects.

### (b) Morphological defects persist in the mature sperm

**Figure 2.**
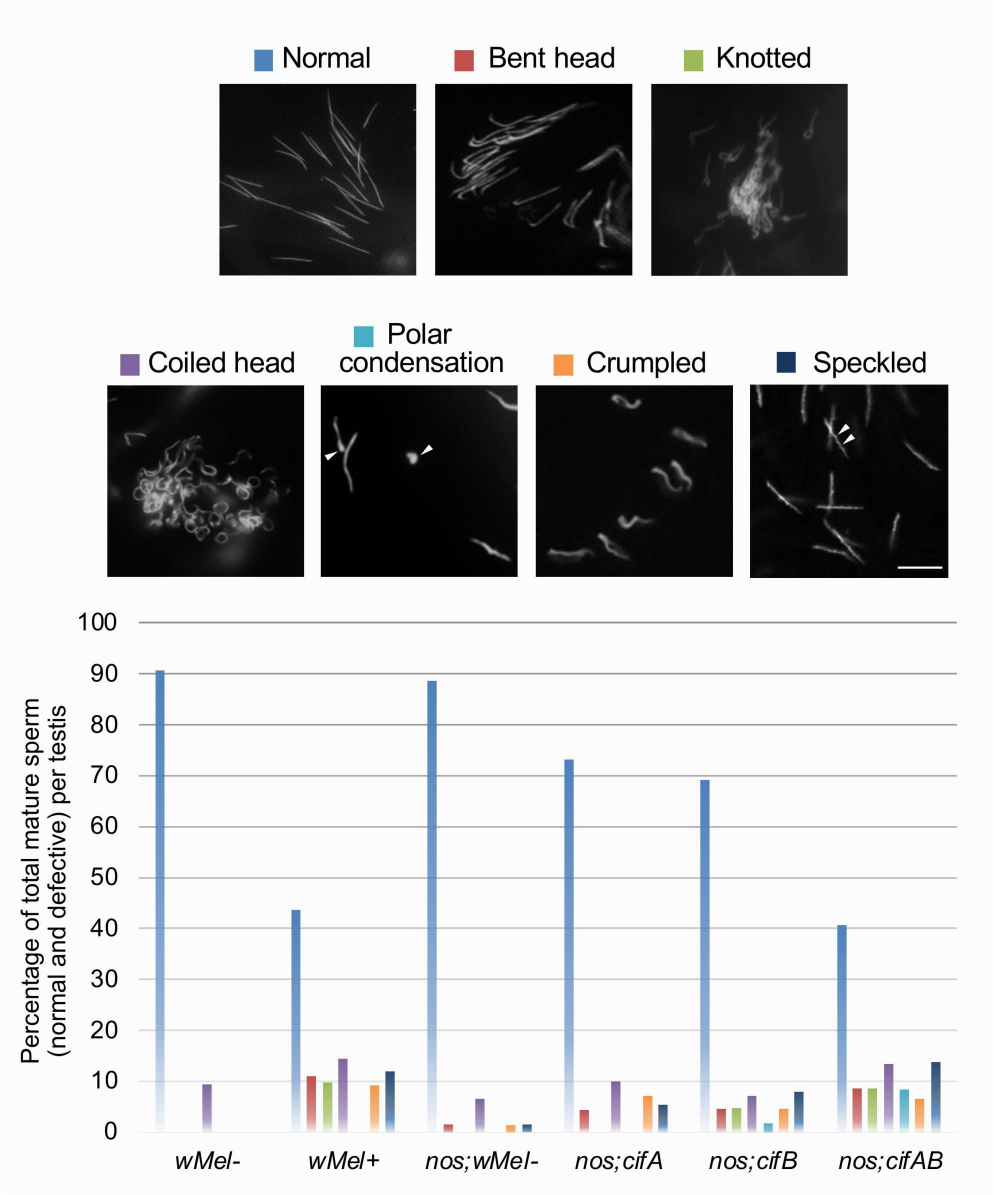
Morphological defects persist in the mature sperm. Testes with seminal vesicles from <8 h old males of CI-causing, wild-type *w*Mel+ and transgenic *cifAB* lines (*n* = 10–15) were dissected and stained with DAPI stain to label mature sperm nuclei. Normal mature sperm morphology is shown compared with defective sperm phenotypes ‘bent head’, ‘knotted,’ ‘coiled head’, ‘polar condensation’, ‘crumpled’ and ‘speckled’. Scale bar: 10 µm. Quantification graph below shows that the defects are predominant in *w*Mel+ and transgenic *cif*-expressing males but are much less frequent or absent in controls.

We further investigated structural disorganization in the mature individualized sperm. While sperm from both *w*Mel− and control transgenic groups showed sharp, thin and straight needle-like structures as expected, the *w*Mel+ and *cif*-expressing males showed defective mature sperm in several different categories defined here as ‘bent head’, ‘knotted’, ‘coiled’, ‘crumpled’, ‘patchy speckled’ and ‘polar condensed’ (figure 2). The knotted phenotype is characterized by deformed sperm heads with a visible knot or loop on one end; the crumpled phenotype appears as wrinkled and wavy; the speckled phenotype is observed as distinct spots or patchy areas within the nucleus due to an unevenly condensed chromatin structure (figure 2, arrowhead); in polar condensed form, the chromatin often is concentrated at one end of the spermatid head (figure 2, arrowhead).

**Figure 3.**
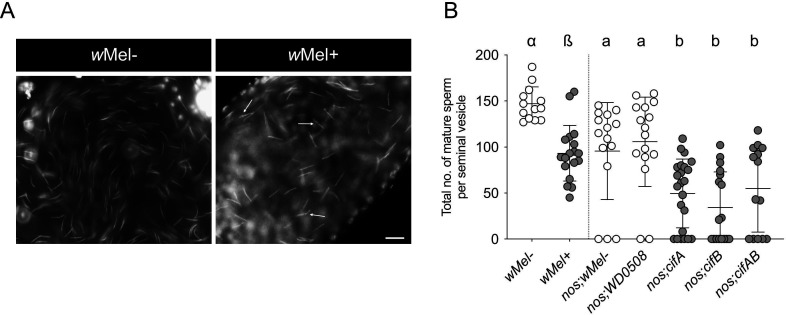
*Wolbachia* and Cifs reduce mature sperm count in the seminal vesicles. (A) Representative image of seminal vesicles (SVs) shows that defective sperm with speckled phenotype (white arrows) transfer in *w*Mel+ SV, whereas normal thin sperm morphology is seen in SV of *w*Mel− males. (B) Total sperm per SV was manually counted from 20 z-stacked images of SVs and graphed. Both *w*Mel+ and transgenic *cif*-expressing males showed significantly less sperm compared with negative controls. Vertical bars represent mean, and error bars represent standard deviation. Scale bar: 10 µm. Letters indicate statistically significant (*p* < 0.05) differences as determined by a pairwise comparison using Mann–Whitney *U*-test between *w*Mel+ and *w*Mel−, and multiple comparisons among transgenic groups based on a Kruskal–Wallis test followed by two-stage step-up method of the Benjamini *et al*. Analysis is performed separately among wild type and transgenic groups (separated by a dotted vertical line) as the experiments were conducted at two separate times. All *p*-values are reported in electronic supplementary material, table S1.

Notably, multiple defective sperm types were observed within a single testis localized at the basal end, right before entering into the seminal vesicle (SV) — the sperm storage organ in males. The one exception was the patchy, speckled sperm phenotype that resides inside the *w*Mel+ SV (*n* = 4/10 SVs examined) compared with thin sperm in *w*Mel− SV (figure 3A). The patchy sperm also infrequently located in a fraction (*n* = 3/10) of spermathecal glands (electronic supplementary material, figure S2, arrows) that serve as conduits for sperm movement between spermatheca (the sperm storage organs in females) and the uterus (the main reproductive chamber). The data suggest that some morphological aberrations render mature sperm incapable to transfer into the seminal vesicle, whereas others can transfer to the female reproductive tract.

Due to the observed shape defects, we hypothesized that the yield of mature sperm in the male’s seminal vesicle may be compromised due to Cif-associated costs. Therefore, we quantified total number of mature sperm per SV in wild type and *cif* transgene-expressing flies. Similar to the spermatid bundle count above in figure 1C, CI-causing *w*Mel+ males had a 36.5% reduced sperm count in the SVs compared with *w*Mel− controls (figure 3B; electronic supplementary material, table S1). Individual and dual *cifAB*-expressing CI males also had 40–65% lower sperm count than their negative control counterpart nos; *w*Mel− and nos; *WD0508* (figure 3B; electronic supplementary material, table S1). This suggests either a delay in mature sperm transfer into the SVs due to slower development, or that the misshaped spermatids fail to mature, resulting in reduced sperm count in the SVs. Contrary to *w*Mel+ wild-type males, we observed several empty SVs (in the range of 25–50%) with no mature sperm in *cif*-expressing transgenic males (figure 3B). This diagnostic difference may be due to the high, constitutive expression of *cif* genes that, in some cases, is able to induce severe damage, resulting in no formation and transfer of mature sperm. As shown in electronic supplementary material, figure S1, *cifAB*-expressing males occasionally produce CI crosses that cannot be rescued by *w*Mel+ females, thereby connecting the association between the null sperm trait of CI and lack of rescue. In summary, *Wolbachia* and Cif proteins adversely impact sperm nuclear shape and counts, thus translating to a compounding set of Cif-associated costs on sperm development.

### (c) Defective needle spermatids and mature sperm suffer from DNA damage

**Figure 4.**
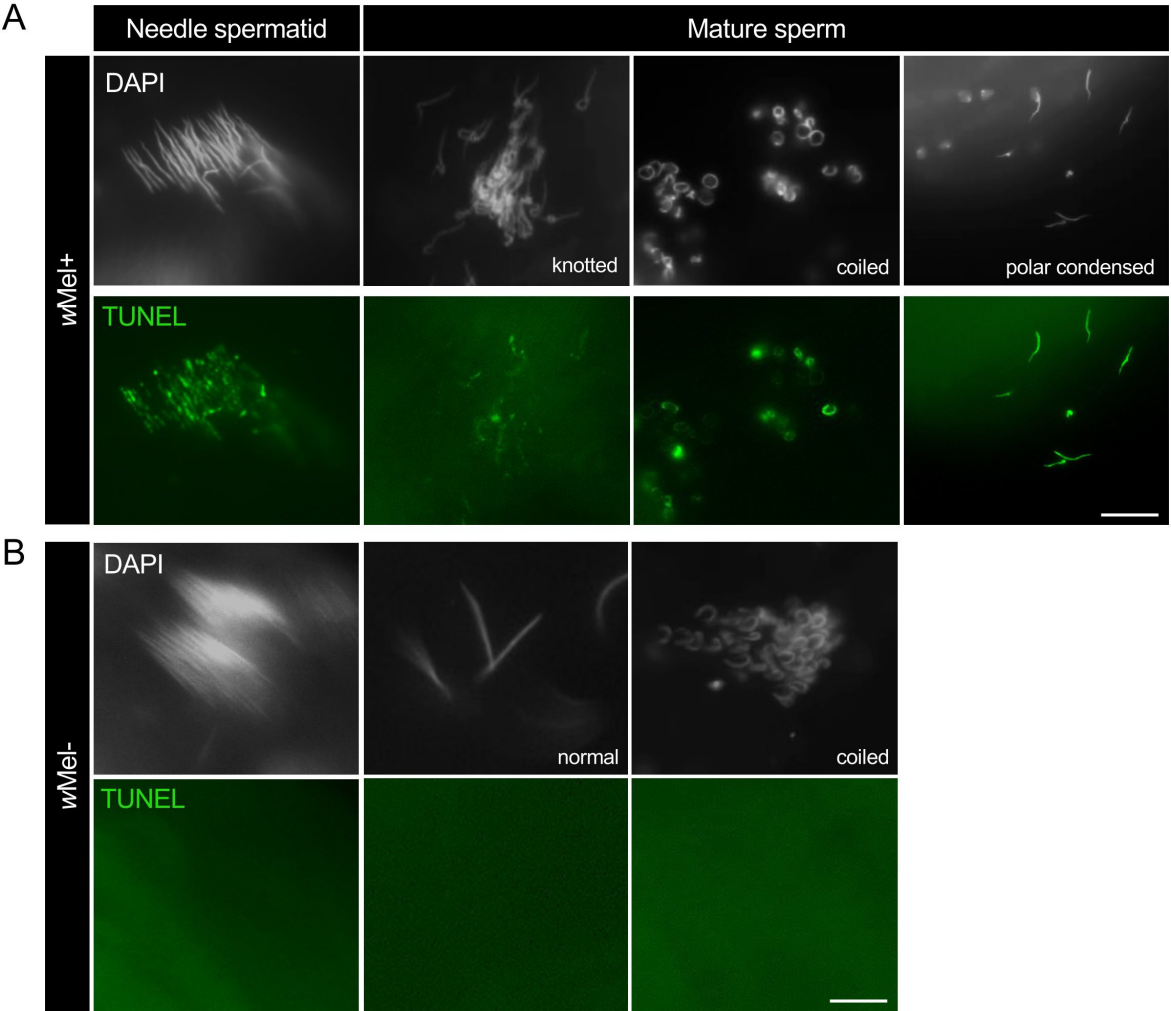
Defective needle spermatids and mature sperm suffer from DNA damage. Terminal deoxynucleotidyl transferase-mediated dUTP nick-end labeling (TUNEL) staining on testes squashes from <8 h old males was performed to visualize DNA breaks in needle spermatids and mature sperm. Compared with *w*Mel− control males, DNA break signals marked by TUNEL (green) were observed in the defective needle-spermatids and mature sperm with knotted, coiled and polar-condensed morphological errors of *w*Mel+ males. DAPI (grey) was used as a control DNA stain. Scale bar: 10 µm.

Abnormal sperm genome packaging and structural defects are often associated with DNA damage across animals [9,10]. To assess whether structural defects observed in maturing sperm make them sensitive to enhanced DNA damage, we used the terminal deoxynucleotidyl transferase-mediated dUTP nick-end labeling (TUNEL) assay, as described previously [7]. In both *w*Mel+ and *cif*-expressing CI males, we only detected DNA damage in misshaped needle spermatids and mature sperm in knotted, coiled and polarly condensed categories (figure 4A). In contrast, there were no DNA damage signals in needle spermatids and mature sperm that did not show shape deformities. In *w*Mel− control males, needle spermatids and mature sperm were normally shaped and devoid of TUNEL signals, as expected. Interestingly, in a few negative control testes with coiled sperm morphology, we did not detect co-staining of DNA with TUNEL (figure 4B), suggesting that despite the morphological aberration, *Wolbachia* and *cif* expression are required to aid in the fragmentation of sperm DNA as reported previously [7].

## 3. Discussion

While the CI adaptation can rapidly spread *Wolbachia* to fixation in arthropod populations, there can be ensuing costs to host sperm biology. In *Drosophila simulans,* for example, *Wolbachia*-carrying symbiotic males develop cysts with abnormal morphology, produce infertile sperm and transfer fewer sperm upon mating [11–16]. *Wolbachia* in *D. melanogaster* also associate with shape defects of mitochondrial derivatives during spermatid elongation [17]. These findings largely predate the discovery of CI-inducing *cif* genes and their functionality, and thus they were unable to evaluate what causal factors underpin these defects. Moreover, it remained unclear if the sperm defects were attributable to *Wolbachia* biology in general or specifically to expression of one or both Cifs. In this study, we report that the costs associated with sperm development, morphology and count in wild-type *D. melanogaster* males are due to CifA and CifB.

During spermiogenesis, the histone-bound paternal DNA is typically replaced by a sperm-specific proteins named protamines [18–20]. This genome-scale repackaging yields a highly compacted and closed chromatin structure that protects the structural integrity of the paternal genome and provides hydrodynamic advantages to the sperm. A defective H–P transition can cause sperm shape defects in *Drosophila* [21–23] and mammals [24,25]. Here, we first show that *w*Mel and Cif proteins initiate structural abnormalities in elongating spermatids at the canoe and needle stages, typically the stages when H–P transition occurs, followed by reduced number of spermatid bundles. Disrupted H–P exchange by *w*Mel and Cifs [6,8] can precipitate into the shape and count defects observed at these stages. Moreover, in *D. melanogaster,* the mitochondrial derivatives play a role in spermatid elongation by providing a structural platform for microtubule reorganization along with supporting the tail elongation [26,27]. Mutant males with poorly segregated mitochondrial derivatives exhibit severe elongation defects [28,29]. *Wolbachia* and CifA localize to the spermatid tail in elongating spermatids [6,8,30], suggesting a putative link that they may interact with mitochondria to disrupt the elongation process.

Further, the shape defects persist in the mature sperm. In *Drosophila*, a set of protamines organize the paternal chromatin into a densely compacted structure to form a sharp and thin needle-like sperm [19,31]. Protamine A and B functionally cooperate with protamine-like Mst77F and Prtl99C proteins for sperm chromatin compaction. Prtl99C is pivotal in mature sperm production and male fertility since a dual knockout of Prtl99C and ProtA/B results in abnormal sperm chromatin compaction relative to the ProtA/B mutant alone [23]. Mst77F incorporates into spermatid nuclei at the H–P transition and cooperates with ProtA/B for proper chromatin condensation and nuclear shape [32]. An additional Mst27D protein is crucial for the formation of microtubules for a normal nuclear elongation [33]. Notably, *Wolbachia* and Cifs alter protein abundance of certain protamines indirectly by upstream depletion of a lncRNA required for a normal H–P transition [7]. Further, several *D. melanogaster* genes are involved in a proper sperm genome packaging and nuclear shaping. For example, *salto* is involved in sperm-head architecture. *Salto*-null males are sterile and show spermiogenesis defects characterized by abnormal nuclear coiling of late spermatids, leading to failure of mature sperm formation [34]. Karyopherins (importins), that mediate protein transport from the cytoplasm into the nucleus, can deposit Mst77F onto the paternal chromatin. Knock out of importins can lead to abnormal nuclear shape and male sterility [35,36]. Notably, some Cif variants can bind karyopherins [37], indicating a functional hypothesis that Cifs may indirectly disrupt nuclear shape by quenching host genetic pathways required for normal sperm architecture and maintenance. Altogether, our findings of malformed mature sperm nuclei conformations in some wild type and transgenic males suggest that *Wolbachia* and Cifs potentially interfere with a variety of sperm-associated proteins to ultimately disrupt normal sperm chromatin architecture.

We previously showed under the Host Modification model of CI that the mature CI sperm in wild type and transgenic males develop with an impaired H–P transition with no malformed nuclear shape [6,8], indicating that despite disrupted chromatin nucleoprotein composition, CI males can be fertile. The CifA enzyme has RNase activity that depletes a lncRNA in primary spermatocytes; and CifA and CifB have modest DNase enzymatic activities that enhance late canoe spermatid DNA damage, both spermatogenic events important for regulating the H–P transition and maintaining sperm nuclear morphology [7,19,22].

The sperm shape and count defects detected here and previously can either be due to *Wolbachia* activities unrelated to CI or parsimoniously to Cif-associated consequences proposed under a ‘Goldilocks zone’, whereby Cifs can either exert not enough, just the right amount, or too much adverse impact on sperm development to render them CI-capable. Insufficient sperm modifications lead to no CI, and hyperactivity of the Cif-induced sperm modifications could tip the balance from conventional CI to sperm defects and infertility. In other words, Cifs’ RNase and/or DNase activity above or below the CI threshold might hypothetically tip the balance between developing sperm that bestow CI versus becoming a malformed sperm. Additionally, the degree of Cif-mediated defects in the H–P transition may surpass the threshold so that the mature sperm develop with abnormal morphology and compromised activity. Indeed, in the mature sperm studied here, we detected DNA damage signals in the morphologically malformed sperm, whereas no signals were observed in the modified mature CI sperm [7].

In addition to the sperm shape errors, we also detected reduced number of mature sperm in the seminal vesicles of wild type and transgenic males. These data validate observations in *D. simulans* where *Wolbachia-*infected males produce approximately 40% less sperm than the uninfected ones [13]. Lower sperm count can have a direct impact on male fitness as high sperm numbers are often important in obtaining successful paternity under sperm competition [38]. Metabolic pathways such as branched-chain amino acid (BCAA) biosynthesis are crucial to sperm development and maturation [39–41]. In the planthopper *Laodelphax striatellus* males, CI-inducing *Wolbachia* can downregulate the expression of various components involved in the BCAA pathway [42]. Thus, it is possible that *w*Mel and Cifs disturb sperm development and fertility by disrupting various metabolic pathway/s.

Our data present the first evidence of a sperm cost linked to the CifA and CifB enzymes from the *w*Mel strain of *Wolbachia* that can have varied impacts on ongoing *Wolbachia*-based vector control strategies, host behaviour and evolutionary biology. For example, Cif-mediated sperm defects can, in theory, lead to reduced reproductive success in released wild type and transgene-expressing males, causing a subsequent reduction in the size of pest or vector populations. However, over time, host populations may evolve resistance or compensatory mechanisms [43]; for example, females might evolve to preferentially mate with uninfected males [44] or develop resistance to the deleterious effects of *Wolbachia* or *cif*-expressing sperm, thus diminishing the long-term effectiveness of population suppression strategies [45]. Moreover, the costs associated with sperm defects may reduce the fitness of *Wolbachia*-infected males, putting them at a disadvantage in direct competition with uninfected males and potentially leading to a decline in population replacement that requires sustainable *Wolbachia* spread in the population [46]. Additionally, the mating competition and dynamics could influence reproductive success within the population, potentially affecting the long-term evolutionary trajectory of host populations, the emergence of new traits or species, etc [47]. In summary, the host modifications of the CifA and CifB proteins from *w*Mel *Wolbachia* are more diverse and consequential than previously known. Disentangling these complex interactions may provide further insight into CI level variation, cell biological cross-talk between the Cifs and the host reproductive system, and evolutionary outcomes between Cifs and different host species spanning basic experimental systems to applied disease vectors.

## 4. Material and methods

### (a) Fly rearing and strains

*Drosophila melanogaster* stocks *y*^1^w^*^ (BDSC #1495), *nos*-GAL4:VP16 (BDSC #4937) and UAS transgenic (TG) lines homozygous for *cifA*, *cifB*, *cifAB* and *WD0508* [4] were maintained at 12:12 light:dark at 25°C and 70% relative humidity on 50 ml of standard cornmeal- and molasses-based food medium. Lines without *Wolbachia* were previously generated [4] through tetracycline treatment for three generations. Wolb_F and Wolb_R3 primers were used to confirm symbiont presence and absence.

### (b) Tissue dissections and morphological defects

To perform morphological assays, we first set up the flies as previously described [48]. Briefly, virginity-controlled wild type (*w*Mel+and *w*Mel−) and *nos*-Gal4:VP16 driver females were aged 9−11 days and mated with TG males [49]. We used *nos*-Gal4:VP16 line since it was previously shown to drive individual and dual *cifAB* expression sufficient to induce near-complete embryonic death [50]. We collected <8 h old wild type and TG Gal4-UAS males as the young, hatched males induce strong CI levels [51,52], anesthetized on ice to stop their movement and dissected whole testes in ice-cold 1× phosphate-buffered saline (PBS) solution. For morphological assays, dissected samples were fixed in 4% paraformaldehyde for 30 min at room temperature followed by three times washing in 1× PBST (1× PBS + 0.1% Triton X-100) before mounting in the vectashield medium containing DAPI. Imaging was performed at 100× magnification using All-in-one Keyence BZ-X700 fluorescence microscope.

For TUNEL assay, dissected testes were treated with 2 mM dithiothreitol for 45 min at room temperature, followed by fixation in 2% paraformaldehyde on ice for 15 min as previously described [7]. After washing in 1× PBS for 2 min, samples were permeabilized in 0.1% Triton X-100 in sodium citrate [10] mg sodium citrate, 10 ml Triton, 10 ml milliQ H2O) for 2 min on ice. After washing in 1× PBS for 2 min, samples were incubated with 50 ml mix of 5 µl enzyme and 45 µl labelling solution (TUNEL *in situ* Cell Death Detection Kit, Fluorescein, Cat. No. 11684795 910 from Roche) for 1.5 h at 37°C in a dark humid chamber. After washing in 1× PBS for 2 min, samples were mounted in the vectashield medium containing DAPI and stored overnight at 4°C. Imaging was performed using green fluorescence filter excited at 488 nm laser for TUNEL and at 359 nm for DAPI stain at 63× magnification using Keyence microscope. Seminal vesicles were imaged with 1 μm z-stacks through the tissue. Images were analysed using ImageJ software. For quantification of abnormalities in sperm DNA morphology, a minimum of 10–20 testes, each from a different male, were analysed per genotype. All spermatid bundles including defective ones were manually scored.

### (c) Hatch rates

Hatch rates were set up as described previously [6]. Briefly, a male and female pair was placed in an 8 oz, round bottom, polypropylene *Drosophila* stock bottle with a grape juice agar plate containing a small amount of yeast placed at the base and secured with tape. These bottles were then placed in a 25°C incubator overnight to allow for courting and mating. The following day, these plates were discarded and replaced with new grape juice agar plates with fresh yeast. After an additional 24 h, the plates were removed and the embryos were counted. The embryo plates were then incubated for 36 h at 25°C before the total number of unhatched embryos were counted. Any crosses with fewer than 25 embryos laid were discarded from the analyses.

### (d) Statistical analysis

All statistical analyses were performed using GraphPad Prism 10 software. While comparing data between two groups, we used a two-tailed, non-parametric Mann–Whitney *U*-test. For comparisons between more than two datasets, we used a non-parametric Kruskal–Wallis one-way analysis of variance test followed by a Dunn’s multiple correction or two-stage step-up method of the Benjamini, *et al.* [53]. This allowed robust testing between all data groups while correcting for multiple test bias. Pairwise χ2 analyses was used to compare defective and normal spermatid bundle categories. All *p*-values are reported in electronic supplementary material, table S1.

## Data Availability

The datasets supporting this article have been uploaded as part of the online supplementary material. Supplementary material is available online [54].
